# Selenium intake and a selenium-centered nutrient pattern are inversely associated with remnant cholesterol among older women in rural China: partial mediation by tumor necrosis factor-α

**DOI:** 10.1186/s12877-025-06807-7

**Published:** 2025-12-23

**Authors:** Baodi Xing, Yu Wang, Jie Yu, Yiwen Liu, Qi Gao, Xinyue Chen, Shuli He, Fan Ping, Lingling Xu, Wei Li, Huabing Zhang, Yuxiu Li

**Affiliations:** 1 Department of Endocrinology, Key Laboratory of Endocrinology of National Health Commission, Translation Medicine Center, Peking Union Medical College Hospital, Chinese Academy of Medical Sciences and Peking Union Medical College, Beijing, China; 2 BeijingChangping District Nankou Community Health Service Center, Beijing, China; 3 Department of Nutrition, Peking Union Medical College Hospital, Chinese Academy of Medical Sciences and Peking Union Medical College, Beijing, China

**Keywords:** Remnant cholesterol, Micronutrient, Selenium, Nutrient pattern, Inflammation, Older adults

## Abstract

**Backgrounds:**

Micronutrients are crucial for metabolic health. Selenium, a vital micronutrient, exerts cardiovascular protection through its anti-inflammatory properties. Remnant cholesterol (RC) is an emerging cardiovascular risk marker, yet the relationship between selenium, nutrient patterns (NPs), and RC remains underexplored, particularly in older people. This cross-sectional study investigates these associations and evaluates the inflammatory marker TNF-α as a potential mediator.

**Methods:**

We analyzed 378 older adults (aged 60–84 years; 55.5% women) from rural northern China, using a 24-hour dietary recall and principal component analysis (PCA) to derive NPs. RC was assessed through a computerized method. Multiple linear regression and restricted cubic splines (RCS) were primarily used to examine the associations, and mediation analysis was used to explore mediating effects, with subgroup analyses to assess sex-specific effects.

**Results:**

Over 80% of participants had inadequate intakes of selenium, vitamin A, vitamins B1/B2/B3, vitamin C, potassium, calcium, and magnesium, with higher prevalence in women. In multiple linear regression, higher selenium intake (quartiles) showed progressively stronger inverse associations with RC and TNF-α (*P* for trend < 0.001 and = 0.016), with more pronounced effects in women. RCS confirmed a linear association with RC in the overall (*P* for nonlinear = 0.058), but no significant relation to TNF-α; in women, both associations were linear. Among four PCA-derived NPs, the “high selenium-vitamin A” pattern showed a significant negative association with RC (β [95% CI]: -0.078 [-0.128, -0.029], *P* = 0.002), particularly in women (β [95% CI]: -0.148 [-0.239, -0.058], *P* = 0.001). TNF-α partially mediated the relationship between selenium and RC in women (indirect effect: -0.002 [-0.0052, -0.0004]), accounting for 15.38% of the total effect.

**Conclusions:**

Selenium intake and a selenium-centered nutrient pattern are inversely associated with RC, with stronger associations in older women. In women, TNF-α partially mediated the association between selenium intake and RC, seeming to suggest a potential inflammation-related pathway. These sex-specific, nutrition-linked correlates of RC may inform geriatric lipid management.

**Supplementary Information:**

The online version contains supplementary material available at 10.1186/s12877-025-06807-7.

## Introduction

As the population ages rapidly, age-related conditions, particularly cardiovascular disease (CVD), are becoming more prevalent, placing considerable burdens on society and the economy [1]. Dyslipidemia is a principal driver of CVD and a core focus of its management. Yet residual risk persists even at guideline-recommended low-density lipoprotein cholesterol (LDL-C) levels, indicating a need for predictors and therapeutic targets beyond LDL-C [2]. Remnant cholesterol (RC), the cholesterol in triglyceride-rich lipoprotein (TRL) remnants, consisting mainly of very-low-density lipoprotein (VLDL) and chylomicron remnants, has emerged as such a candidate [3]. Large Mendelian randomization studies and prospective cohorts link higher RC with increased CVD risk independent of LDL-C [3, 4]. Furthermore, a large retrospective study found that long-term cumulative RC exposure was associated with a higher risk of major adverse cardiovascular events in patients aged ≥ 75 years with atherosclerotic cardiovascular disease [5]. These findings support RC as a potential lipid-lowering target and underscore the need to identify modifiable determinants to maintain optimal RC levels.

Dietary interventions, particularly maintaining adequate micronutrient intakes, are important to managing dyslipidemia and CVD. Some micronutrients, such as dietary vitamin E, have been reported to be inversely associated with RC [6]. Selenium, that is an essential micronutrient with anti-inflammatory, antioxidant, and anti-apoptotic properties, has been linked to lower circulating lipids and reduced CVD incidence and mortality [7, 8]; however, its relationship with RC has not been examined. Mechanistically, selenium repletion has been shown to prevent the increase in hepatic VLDL secretion observed under selenium deficiency [9]. Hepatic clearance of TRL remnants depends on receptors such as low-density lipoprotein receptor (LDLR) (and related pathways), and selenium deficiency can downregulate LDLR, suggesting that adequate selenium may help maintain remnant clearance in the liver [10]. Together, these findings provide a plausible route by which higher selenium intake may lower RC.

Moreover, because single-nutrient analyses may fail to capture the overall effects of diet; in contrast, dietary pattern approaches account for complex interactions between nutrients, which can provide more practical guidance for nutritional interventions. Principal component analysis (PCA) is a data-driven method that reduces the dimensionality of correlated foods or nutrients to identify characteristic dietary components and assess their associations with health outcomes [11]. Nonetheless, evidence relating PCA-derived nutrient patterns (NPs) to RC remains limited.

Inflammation is a pivotal pathway through which RC drives CVD and other disorders. The cardioprotective effects of several micronutrients, for example selenium, are likewise attributed to their anti-inflammatory properties. Tumor necrosis factor-α (TNF-α), a major pro-inflammatory cytokine, is central to the link between suboptimal diets and metabolic dysregulation. Intake of nutrients such as vitamins C and E has been shown to lower serum TNF-α and improve lipid profiles [12], suggesting that TNF-α could mediate nutrition–lipid relationships. Direct evidence for TNF-α as a mediator between micronutrients or nutrient patterns and RC, however, remains limited.

Thus, using data from a rural Chinese cohort of older adults, we aim to investigate whether selenium and other micronutrients, individually and as PCA-derived nutrient patterns, are associated with RC, and evaluated TNF-α as a potential mediator.

## Materials and methods

### Study population

This study utilized baseline data from an ongoing community-based cohort in Changping District, Beijing, China, collected from March 2014 to July 2021. The cohort initially comprised 480 residents aged ≥ 60 years. Each participant completed a structured questionnaire (including sociodemographic factors, habitual diet, medical history, medication use), followed by a standardized physical examination and fasting blood sampling.

Participants were excluded based on the following criteria: (i) severe cardiovascular diseases, significant liver or renal insufficiency, or chronic gastrointestinal disorders (*n* = 8); (ii) missing metabolic data, such as lipid profiles (*n* = 21); (iii) use of nutrient supplements or medications (e.g., lipid-lowering or antidiabetic drugs) affecting serum lipids within the preceding 3 months (*n* = 20); (iv) unavailable dietary data or inappropriate energy intake (< 500 kcal or >5000 kcal) [13] (*n* = 53). Ultimately, 378 eligible participants aged 60–84 years were included in the analysis. All individuals voluntarily signed informed consent prior to inclusion in the study. This study was approved by the Ethics Committee of Peking Union Medical College Hospital.

### Clinical assessment

All participants were measured for height, weight, waist circumference (WC), systolic blood pressure (SBP), and diastolic blood pressure (DBP), as detailed in previous studies [14]. Body mass index (BMI) was calculated as body weight divided by height squared (kg/m^2^). According to standard measurements, hypertension (HTN) is defined as SBP ≥ 140mmHg and/or DBP ≥ 90mmHg and/or individuals currently taking anti-hypertensive medications.

### Biochemical analysis

Venous blood samples were collected after an overnight fast of more than 10 h. Fasting and 2-hour post-load blood glucose, insulin, and C-peptide levels were measured oral glucose tolerance trial (OGTT). Each subject was classified into normal glucose tolerance (NGT), pre-diabetes mellitus (preDM), or diabetes mellitus (DM) based on their glucose levels [15]. Glycosylated hemoglobin (HbA1c) levels were analyzed by high-performance liquid chromatography (HPLC, intra-assay coefficient of variation (CV) < 3%, inter-assay CV < 10%). Fasting plasma glucose (FPG), alanine aminotransferase (ALT), serum creatinine (sCr), serum uric acid (sUA), and lipid profiles, including triglyceride (TG), total cholesterol (TC), LDL-C and high-density lipoproteins cholesterol (HDL-C), were evaluated by an automated analyzer (AU5800 automatic biochemical analyzer, Beckman Coulter). The estimated glomerular filtration rate (eGFR) was accessed using the Chronic Kidney Disease Epidemiology Collaboration equation [16].

RC was defined as TC minus HDL-C minus LDL-C. The LDL-C levels were calculated based on TC, HDL-C, and TG levels according to the Friedewald formula unless TG was significantly elevated (>4 mmol/L) [17, 18]. According to the Chinese Guidelines for Lipid Management [19], dyslipidemia is diagnosed if TC ≥ 6.2mmol/L and/or LDL-C ≥ 4.1mmol/L and/or HDL-C < 1.0mmol/L and/or TG ≥ 2.3mmol/L and/or a prior hospital diagnosis of hyperlipidemia without current lipid-lowering treatment.

Serum tumor necrosis factor α (TNFα) levels were determined by using enzyme- linked immunosorbent assay (ELISA, Cloud-Clone Corp., Houston, TX, USA).

### Dietary assessment

Given the limited educational attainment and relatively simple dietary habits of this rural older population, dietary intake was assessed using a 24-hour dietary recall [20]. The dietary data were reviewed by professional clinical nutritionists and entered into nutrition calculation software (developed by researchers based on the Microsoft Office Access 2007 database). Nutrient composition was derived from the China Food Composition Table (2009) database [21]. Prioritizing computability and data completeness, and consistent with routine assessment in Chinese clinical nutrition practice (in alignment with national dietary guidance) [22], we focused on total energy and 15 commonly assessed micronutrients: total vitamin A, vitamins B1, B2, B3, C, and E; phosphorus, potassium, calcium, magnesium, zinc, iron, selenium, copper, and manganese.

### Principal component analysis-derived dietary patterns

All micronutrient intakes were energy-adjusted with the residual method before pattern extraction [23]. Suitability for PCA was confirmed by a Kaiser–Meyer–Olkin (KMO) value of 0.846 (criterion: KMO >0.70, high values closer to 1 indicate stronger correlations) and a significant Bartlett’s test of sphericity (*P* < 0.001; criterion: *P* < 0.05). The number of NPs (or factor) was determined according to eigenvalues >1.0, scree-plot inflection, and interpretability. Varimax rotation was applied using the maximum variance method to obtain nutrient-based factor loadings. Absolute value of factor loadings ≥ 0.47 were deemed major contributors and used to name the NPs [24]. A negative loading indicates lower intake, whereas a positive loading indicates higher intake. Within a specific nutrient pattern, higher factor scores indicate a greater adherence to that pattern.

### Statistical analysis

Baseline characteristics were compared by sex because metabolic profiles and micronutrient intakes differ between men and women. Given potential comorbidities and differences in nutrient absorption among older adults, we defined nutrient inadequacy as intake below the recommended nutrient intake (RNI) in the Chinese Dietary Guidelines [22] and compared prevalence by sex. All variables were first assessed for normality. Continuous data were reported as mean ± standard deviation (SD) when normally distributed and as median (interquartile range) when skewed; group differences were tested with the independent-samples t-test or the Mann-Whitney U-test, respectively. Categorical variables were expressed as percentages and compared with the chi-square test. Pairwise Pearson correlations were used to assess associations between each micronutrient intake (energy-adjusted via the residual method) and RC and TNF-α. Multivariable linear regression then examined selenium intake, modeled both continuously and by quartiles (Q1 reference), in relation to RC and TNF-α, adjusting for age, sex, total energy intake, WC, DM, HTN, dyslipidemia, ALT, and eGFR. Furthermore, restricted cubic splines (RCS) with knots at the 5th, 35th, 65th, and 95th percentiles were utilized to determine the nonlinearity in the associations of selenium intake with RC and TNF-α. Subsequently, associations of PCA-derived NP scores with RC and TNF-α were evaluated using multiple linear regression with the same covariate adjustment. Sex-stratified linear regression, RCS, and mediation analyses were also performed.

The mediating role of TNF-α in the associations of individual selenium intake and NP scores with RC were evaluated using the PROCESS macro (version 3.4) for SPSS. Indirect effects were estimated with 5,000 bias-corrected bootstrap resamples to generate 95% confidence intervals (CIs). Mediation was considered significant when the CI excluded zero.

Statistical analyses were performed using SPSS Statistics 26.0 (IBM Corp., Chicago, IL, USA) and R 4.2, and a two-sided *P*-value < 0.05 was considered statistically significant.

## Results

### Comparison of basic and metabolic characteristics in women and men

Among the 378 participants, the mean age was 65.98 ± 5.19 years with no sex difference. Compared with men, women had higher RC, TC, TG, HDL-C, and BMI, as well as a higher prevalence of HTN and dyslipidemia (all *P* < 0.05). In contrast, sUA and eGFR were lower in women (both *P* < 0.05). FPG, 2hPG, HbA1c, the prevalence of diabetes, ALT, AST, and TNF-α did not differ between sexes (all *P* > 0.05) (Table 1).

**Table 1:** The basic and metabolic characteristics between different sexes

	Overall(*n* = 378)	Women(*n* = 210)	Men(*n* = 168)	*P*
Age (y)	65.98 ± 5.19	65.88 ± 5.02	66.11 ± 5.41	0.666
BMI (kg/m^2^)	25.78 ± 3.35	26.19 ± 3.28	25.28 ± 3.37	0.009*
WC (cm)	90.21 ± 10.17	89.36 ± 9.36	91.27 ± 11.05	0.074
SBP (mmHg)	134.34 ± 17.16	135.23 ± 16.79	133.22 ± 17.60	0.26
DBP (mmHg)	78.31 ± 11.65	77.63 ± 11.36	79.16 ± 11.98	0.206
TC (mmol/L)	5.04 ± 1.14	5.21 ± 1.21	4.83 ± 1.02	0.001*
TG (mmol/L)	1.33 (0.93, 2.00)	1.60 (1.11, 2.22)	1.15 (0.83, 1.79)	< 0.001*
HDL-C(mmol/L)	1.26 ± 0.33	1.29 ± 0.36	1.22 ± 0.29	0.039*
LDL-C(mmol/L)	2.84 ± 0.81	2.89 ± 0.81	2.77 ± 0.80	0.13
RC (mmol/L)	0.96 ± 0.53	1.05 ± 0.54	0.85 ± 0.49	< 0.001*
FPG (mmol/L)	6.46 (5.70, 8.00)	6.44 (5.77, 8.00)	6.47 (5.60, 8.00)	0.851
2hPG (mmol/L)	10.40 (7.26, 17.60)	10.70 (7.44, 17.60)	10.20 (6.83, 17.50)	0.441
HbA1c (%)	6.54 ± 1.45	6.57 ± 1.46	6.51 ± 1.45	0.711
ALT (U/L)	19.35 (15.00, 25.98)	20.00 (15.07, 27.00)	19.00 (14.95, 24.00)	0.138
sUA (umol/L)	299.25 ± 77.63	285.67 ± 73.97	316.24 ± 78.97	< 0.001*
eGFR (mL/min/1.73m^2^)	88.89 ± 20.48	85.46 ± 15.28	93.16 ± 24.91	< 0.001*
TNF-α (pmol/L)	23.90 ± 11.17	24.4 ± 11.30	22.96 ± 11.00	0.401
HTN (%)	212 (56.1)	131 (62.4)	81 (48.2)	0.008*
Dyslipidemia (%)	198 (52.4)	122 (58.1)	76 (45.2)	0.017*
Glucose state (%)				0.303
NGT	85 (22.8)	41 (20.0)	44 (26.3)	
PreDM	77 (20.7)	46 (22.4)	31 (18.6)	
DM	210 (56.5)	118 (57.6)	92 (55.1)	

*Abbreviation*: *BMI* body mass index, *WC* waist circumference, *SBP* systolic blood pressure, *DBP* diastolic blood pressure, *TC* total cholesterol, *TG* triglyceride, *HDL-C* high-density lipoprotein cholesterol, *LDL-C* low-density lipoprotein cholesterol, *RC* remnant cholesterol, *FPG* fasting plasma glucose, *2hPG* OGTT 2 h plasma glucose, *HbA1c* glycated hemoglobin, *HTN* hypertension, *ALT* alanine aminotransferase, *sUA* serum uric acid, *eGFR* estimated glomerular filtration rate, *TNF-α* tumor necrosis factor-α, *NGT* normal glucose tolerance, *preDM* prediabetes mellitus, *DM* diabetes mellitus

*P < 0.05 means statistical difference

### Comparison of micronutrient intakes between women and men

Women showed lower total energy intake and significantly lower intakes of B vitamins (B1, B2, B3), vitamin E, and several minerals, including phosphorus, potassium, magnesium, iron, selenium, zinc, copper, and manganese, than men (all *P* < 0.05). Intakes of vitamins A and C and calcium did not differ by sex (*P* > 0.05).

Inadequate intake (< RNI) was common in the cohort: more than 80% of participants were below the RNI for vitamin A, vitamins B1/B2/B3, vitamin C, potassium, calcium, magnesium, selenium, and zinc, and over 50% were below the RNI for phosphorus and manganese. Compared with men, women had a higher prevalence of inadequacy for vitamin B3, phosphorus, magnesium, selenium, and manganese, but a lower prevalence for vitamin A; inadequacy for other micronutrients was similar between sexes (Table S1 and S2).

### Correlation analysis between micronutrient intakes and RC as well as TNF-α

In the overall sample, vitamins B2 and B3, phosphorus, and selenium were inversely correlated with RC (*r* = −0.145, −0.135, −0.115, and − 0.183, respectively; all *P* < 0.05). For TNF-α, vitamin E (*r* = −0.334) and selenium (*r* = −0.180) showed inverse correlations (both *P* < 0.05) (Table 2).

**Table 2:** Pearson correlation analysis between micronutrient intakes and RC as well as TNF-α in the population

	RC						TNF-α					
	overall		women		men		overall		women		men	
	ρ	*P*	ρ	*P*	ρ	*P*	ρ	*P*	ρ	*P*	ρ	*P*
Vitamin A	0.047	0.360	−0.017	0.804	0.133	0.086	0.118	0.138	0.111	0.278	0.121	0.350
Vitamin B1	0.036	0.489	0.084	0.228	0.076	0.325	0.118	0.139	0.117	0.250	0.167	0.194
Vitamin B2	**−0.145**	0.005*	**−0.137**	0.047*	−0.120	0.121	−0.147	0.063	**−0.238**	0.018*	0.009	0.944
Vitamin B3	**−0.135**	0.008*	**−0.156**	0.023*	−0.100	0.197	0.020	0.798	−0.031	0.759	0.093	0.474
Vitamin C	−0.041	0.422	**−0.142**	0.040*	0.013	0.867	−0.119	0.135	−0.035	0.733	−0.193	0.133
Vitamin E	0.045	0.386	0.033	0.636	0.051	0.510	**−0.334**	< 0.001*	**−0.339**	0.001*	**−0.335**	0.008*
Phosphorus	**−0.115**	0.026*	**−0.143**	0.039*	−0.114	0.142	−0.047	0.555	−0.049	0.633	−0.054	0.678
Potassium	−0.045	0.388	−0.113	0.101	−0.006	0.940	−0.063	0.428	−0.039	0.701	−0.092	0.477
Calcium	−0.068	0.188	−0.112	0.106	−0.054	0.489	−0.122	0.126	−0.079	0.438	−0.177	0.169
Magnesium	−0.092	0.074	**−0.137**	0.047*	−0.090	0.244	−0.031	0.696	−0.021	0.837	−0.056	0.665
Iron	−0.090	0.080	**−0.172**	0.013*	−0.004	0.958	−0.105	0.186	−0.128	0.210	−0.082	0.525
Selenium	**−0.183**	< 0.001*	**−0.223**	0.001*	**−0.165**	0.033*	**−0.180**	0.023*	**−0.287**	0.004*	−0.051	0.694
Zinc	−0.081	0.114	**−0.150**	0.029*	−0.033	0.667	−0.077	0.333	−0.067	0.510	−0.094	0.466
Copper	0.018	0.734	−0.013	0.854	−0.001	0.987	−0.012	0.881	−0.030	0.770	0.011	0.933
Manganese	0.037	0.477	−0.034	0.621	0.081	0.299	0.096	0.229	−0.034	0.621	0.029	0.822

Statistically significant micronutrients are shown in bold

*P < 0.05 means statistical difference

In sex-stratified analyses, the correlation between selenium intake and RC persisted in both women and men, with a stronger association in women (women: *r* = −0.223; men: *r* = −0.165; both *P* < 0.05). In women, RC also correlated with vitamin B2, vitamin B3, vitamin C, phosphorus, magnesium, iron, and zinc (all *P* < 0.05), whereas in men no correlations between additional micronutrient and RC were observed. Across sexes, vitamin E remained inversely correlated with TNF-α (women: *r* = −0.339; men: *r* = −0.335; both *P* < 0.05); additionally, vitamin B2 and selenium were inversely correlated with TNF-α in women only (both *P* < 0.05) (Table 2).

### Associations of selenium intake with RC and TNF-α in multivariable linear regression and restricted cubic splines

In unadjusted models (model 1), selenium intake (continuous) was inversely associated with both RC and TNF-α (β = −0.005 and − 0.160; *P* < 0.001 and *P* = 0.023, respectively). After adjusting for confounders (model 2), the association with RC remained significant (β = −0.003; *P* = 0.013), whereas the association with TNF-α was attenuated and no longer significant (β = −0.142; *P* = 0.067). When modeled by quartiles (Q1 reference), higher selenium intake was associated with monotonic decreases in RC and TNF-α (*P* for trend < 0.001 and 0.016, respectively) (Table 3).

**Table 3:** Multiple linear regression of selenium intakes with RC and TNF-α

	model 1		model 2	
RC	β(95%CI)	*P*	β(95%CI)	*P*
1 unit increment	−0.005(−0.008,−0.002)	< 0.001*	−0.003(−0.006,−0.001)	0.013*
Q1 (≤ 20.97ug)	1(ref)		1(ref)	
Q2 (20.97–29.97.23ug)	−0.070(−0.219,0.079)	0.355	−0.020(−0.155,0.116)	0.774
Q3 (29.23–37.23.90ug)	−0.251(−0.400,−0.103)	0.001*	−0.200(−0.341,−0.058)	0.006*
Q4 (> 37.90ug)	−0.348(−0.497,−0.199)	< 0.001*	−0.272(−0.409,−0.134)	< 0.001*
*P* for trend	< 0.001*		< 0.001*	
TNF-α				
1 unit increment	−0.160(−0.297,−0.022)	0.023*	−0.142(−0.294,0.010)	0.067
Q1 (≤ 20.97ug)	1(ref)		1(ref)	
Q2 (20.97–29.97.23ug)	1.569(−2.570,5.708)	0.455	1.193(−3.213,5.599)	0.593
Q3 (29.23–37.23.90ug)	−4.379(−9.355,0.596)	0.084	−4.406(−9.896,1.083)	0.115
Q4 (> 37.90ug)	−6.810(−11.920,−1.700)	0.009*	−6.241(−11.758,−0.725)	0.027*
*P* for trend	0.003*		0.016*	

*Model1* crude model, *model2* adjusted age, sex, total energy intake, WC, DM, HTN, dyslipemia, ALT, and eGFR

*P < 0.05 means statistical difference

**Fig. 1:**
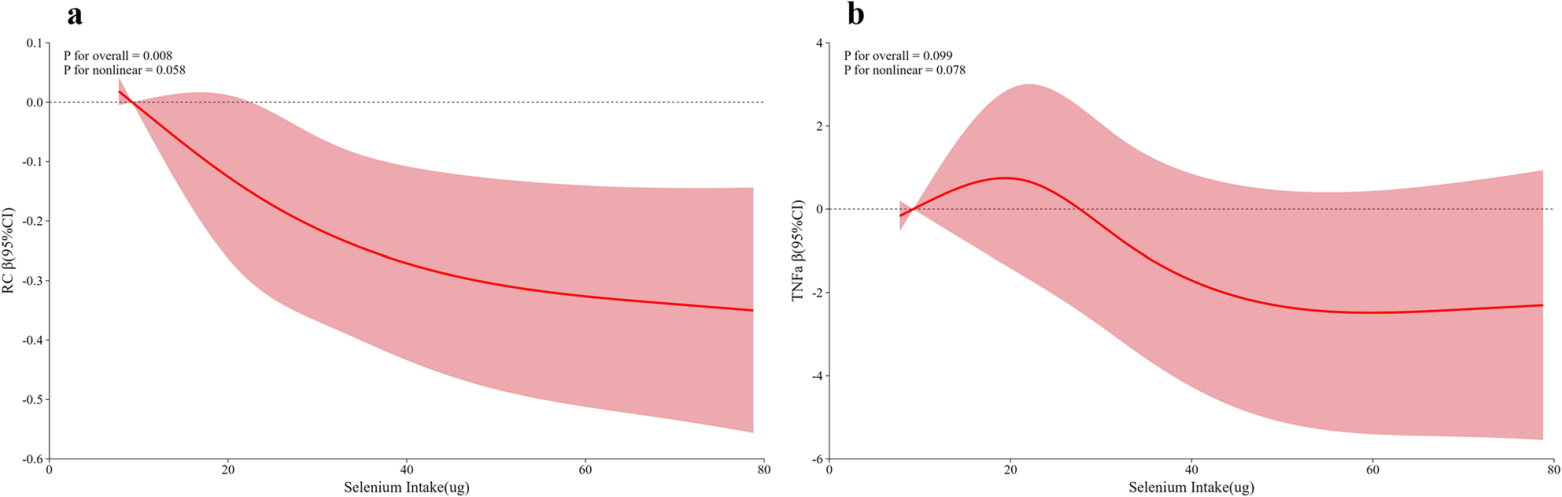
The real relationship between selenium intake and remnant cholesterol (**a**) and tumor necrosis factor α (**b**) through restricted cubic spline.** |**The model was adjusted for age, sex, total energy intake, DM, HTN, dyslipidemia, ALT, and eGFR

Furthermore, RCS supported a linear association between selenium intake and RC (*P* for overall = 0.008, *P* for nonlinear = 0.058). For TNF-α, neither the overall association nor nonlinearity was significant (*P* for overall = 0.099, *P* for nonlinear = 0.078) (Fig. 1).

**Fig. 2:**
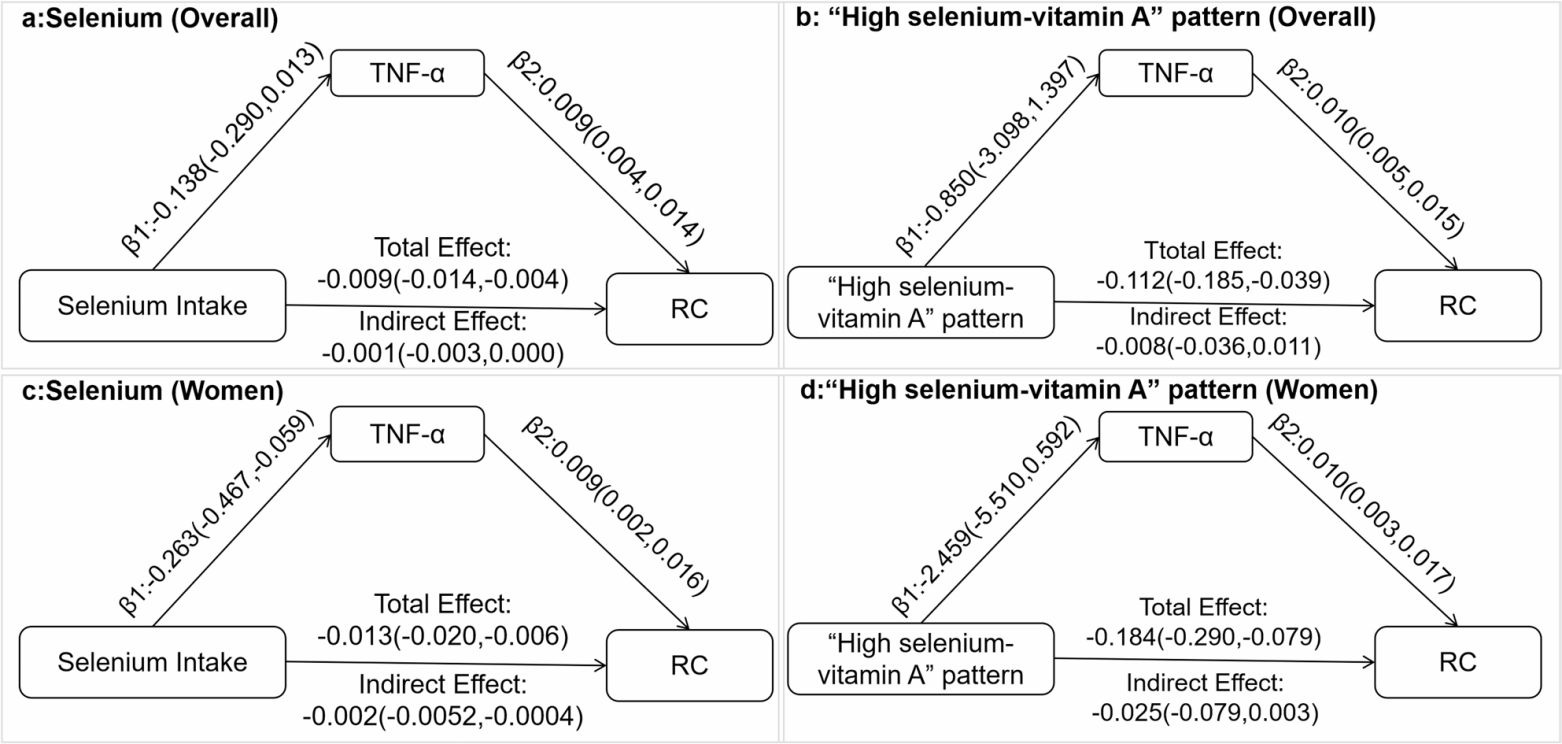
The mediated effect of TNF-α on the correlation between selenium intake or “selenium-vitamin A” pattern and remnant cholesterol. |**a** and **c**: the mediation effect of TNF-α on selenium in overall and female, respectively; **b** and **d**: the mediation effect of TNF-α on “selenium-vitamin A” pattern in overall and female, respectively. The model was adjusted for age, sex, total energy intake, DM, HTN, dyslipidemia, ALT, and eGFR

### Four main nutrient patterns derived by PCA

We identified four nutrient patterns through PCA, which accounted for 68.97% of the total variance in micronutrient intakes. These NPs were named based on the factor loadings of nutrients, as indicated in Table S3 and Fig. S1. The first NP exhibited high positive loadings of vitamin C, vitamin B3, and eight minerals except selenium; accordingly, it was termed the “High vitamin C/B3-multiple minerals” pattern. The second NP was characterized by increased positive loadings of vitamin B1/B2, and thus named the “High vitamin B1/B2” pattern. The third NP exhibited a strong negative loading for vitamin E and was labeled the “ Low vitamin E” pattern. The fourth nutrient pattern is defined by high selenium intake. Vitamin A had the second-highest loading in this pattern, higher than in the other patterns, though below the 0.47 (0.40, near-threshold), supporting the “selenium-vitamin A” label.

### The associations of four nutrient patterns with RC and TNF-α in multivariate linear regression

In unadjusted models (model 1), only the “ high selenium-vitamin A” pattern was inversely associated with RC (β[95%CI]: −0.106[−0.160, −0.053]; *P* < 0.001). This association remained significant after multivariable adjustment (model 2) (β[95%CI]: −0.078[−0.128, −0.029]; *P* = 0.002). No other nutrient pattern showed a significant association with RC, and none of the patterns were associated with TNF-α (Table 4).

**Table 4:** The linear regression of four nutrient patterns with RC and TNF-α

	High vitamin C/B3-multiple minerals		High vitamin B1/B2		Low vitamin E		High selenium-vitamin A	
RC	β(95%CI)	*P*	β(95%CI)	*P*	β(95%CI)	*P*	β(95%CI)	*P*
model 1	−0.027 (−0.081,0.028)	0.332	−0.014 (−0.068,0.041)	0.624	0.009 (−0.046,0.063)	0.758	−0.106 (−0.160,−0.053)	< 0.001*
model 2	−0.002 (−0.048,0.051)	0.936	−0.028 (−0.094,0.038)	0.358	−0.011 (−0.060,0.037)	0.653	−0.078 (−0.128,−0.029)*	0.002*
TNF-α								
model 1	−0.860 (−2.187,0.466)	0.202	0.805 (−1.092,2.702)	0.403	3.654 (2.060,5.248)	< 0.001*	−0.913 (−2.906,1.081)	0.367
model 2	−0.538 (−2.040,0.964)	0.480	2.517 (−0.351,5.385)	0.085	3.733 (2.082,5.384)	< 0.001*	−0.980 (−3.238,1.279)	0.393

*Model1* crude model, *model2* adjusted age, sex, total energy intake, WC, DM, HTN, dyslipemia, ALT, and eGFR

*P < 0.05 means statistical difference

### Subgroup analysis of linear regression and restricted cubic splines based on sex

In sex-stratified models, selenium intake, modeled continuously, was significant only in women (*P* = 0.001). When by quartiles, it was inversely associated with RC in both sexes, with a progressively stronger inverse trend across quartiles (*P* for trend < 0.001 for women and = 0.037 for men), and was more prominent in women. By contrast, selenium intake showed an inverse association with TNF-α only in women (*P* = 0.012). (Table S4) RCS further indicated that, in women, both associations were linear (RC: *P* for overall = 0.020, *P* for nonlinear = 0.099; TNF-α: *P* for overall = 0.029, *P* for nonlinear = 0.387), whereas in men neither association was significant (RC: *P* for overall = 0.203, *P* for nonlinear = 0.153; TNF-α༚*P* for overall = 0.783, *P* for nonlinear = 0.606) (Fig. S2).

For NPs, the “high selenium-vitamin A” pattern was negatively associated with RC in women (β = −0.148; *P* = 0.001) but not in men (β = −0.034; *P* = 0.240). No other NPs were associated with RC in either sex. Regarding TNF-α, the “high vitamin B1/B2” pattern was positively associated in men (*P* = 0.027), whereas the “ low vitamin E” pattern was inversely associated in both sexes (*P* = 0.002 for women and = 0.003 for men); the selenium–vitamin A pattern was not associated with TNF-α in either sex (Table S5).

We also tested sex interactions for selenium intake and the “high selenium-vitamin A” pattern with RC and TNF-α. The interaction between selenium and sex was significant for RC (*P* for interaction = 0.023) but not for TNF-α (*P* = 0.113). No significant interactions for the pattern and sex were observed for either RC (*P* = 0.093) or TNF-α (*P* = 0.202). In simple-slope analyses, selenium intake showed significant inverse slopes in women for both RC and TNF-α, whereas slopes in men were smaller and not significant. For the pattern, the inverse slope for RC was evident in women but not in men, whereas slopes for TNF-α were not significant in either sex. These patterns are consistent with the subgroup analyses and indicate a more pronounced association in women. (Fig.S2 and Table S6)

### The mechanism exploration of TNF-α on the relationship between selenium intake or“high selenium-vitamin A” pattern and RC

Previous findings indicated that selenium was negatively correlated with both RC and TNF-α in the overall and among women, prompting an investigation into whether TNF-α mediates the relationship between the selenium intake or “selenium-vitamin A” pattern and RC. Mediation analysis was thus conducted in the overall population and female subgroup. The results indicated that TNF-α mediate the relationship between selenium intake and RC only in women, accounting for approximately 15.38% of the association. However, no mediating effect of TNF-α was observed in the relationship between the “selenium-vitamin A” pattern and RC (Fig. 2 ).

## Discussion

In this community-based cohort of older adults in rural northern China, we observed low adequacy of micronutrient intakes, particularly selenium, with lower adequacy in women. Individual selenium intake showed a linear and inverse association with RC, especially in women; among four PCA-derived nutrient patterns, only the “high selenium-vitamin A” pattern related inversely to RC, predominantly in women. In mediation analyses, TNF-α accounted for a modest proportion of the association between selenium and RC in women, whereas no mediation was detected for the nutrient pattern itself. These data suggest a sex-specific nutritional correlation of lower RC and point to inflammation as a plausible pathway in women.

RC is increasingly recognized as a clinically informative lipid parameter linked to cardiovascular risk. Large cohort and Mendelian randomization studies show that higher RC is associated with greater risks of CVD and related adverse outcomes, even after adjustment for LDL-C [3–5], supporting greater clinical consideration of RC as part of comprehensive lipid assessment and management. In our cohort, women exhibited higher RC and lower selenium adequacy, underscoring the need to identify modifiable, diet-related correlates of RC, particularly in older women.

Selenium is an essential micronutrient linked to lipid metabolism and cardiovascular risk. Observational studies report inverse associations of dietary selenium with TC, LDL-C, and TG, and positive correlations of serum selenium with HDL-C [25, 26]. In U.S. adults, moderate dietary selenium intake is associated with lower CVD risk, particularly in participants aged 50 and above [8]. A study in patients with nonalcoholic fatty liver disease further suggested a U-shaped relation between selenium intake and cardiovascular mortality, with the lowest risk around 104.1–150.6 µg/day and higher risk outside this range [27]. Focusing on RC, we observed inverse associations with both selenium intake and a selenium-centered nutrient pattern, with the strongest inverse association at intake ≥ 37.9 µg/day (Q4 vs. Q2 vs. Q3: β = −0.272 vs. −0.02 vs. −0.200), supporting consideration of selenium adequacy as part of nutrition-oriented strategies targeting RC. RCS further indicated that this association was linear. Accumulating evidence suggests that the health effects of selenium intake may be bidirectional, with potential harm at both insufficient and excessive intakes [28]. Currently, selenium intake varies widely (~ 93–134 µg/day in the United States; ~40 µg/day in parts of Europe) [29]. In China, despite a recommended intake of 60 µg/day, our cohort averaged 26.32 µg/day with a high prevalence of selenium inadequacy (93.4%). This is similar to a national survey of Chinese adults aged ≥ 60 years [30], which reported a mean intake of 34.6 µg/day and 81.1% of participants below the estimated average requirement. The principal dietary sources of selenium are nuts, seafood, and meat (including offal) [28]. However, in our rural northern China cohort, the predominant source was refined grains (45.6%), followed by meat (19.3%, mainly pork) and eggs/diaries (16.1%) with nuts/legumes only 2.6% (see Fig. S4). The source pattern, together with our use of the more conservative RNI threshold likely account for the difference. These consistent findings underscore the importance of maintaining adequate selenium intake in older adults.

It is now suggested that selenium and selenoproteins may exert cardioprotective effects by dampening inflammation, mitigating ROS-driven oxidative stress, improving endoplasmic reticulum stress, and reducing hepatic lipid deposition, with inflammation representing a key axis [29, 31, 32]. A meta-analysis of population studies reported that selenium supplementation was associated with lower circulating CRP [33], and preclinical work indicates that selenium-rich diets enhance selenoprotein activity that inhibits NF-κB signaling, thereby reducing the release of pro-inflammatory cytokines such as TNF-α, interleukin-1β (IL-1β), and IL-6 [34]. Consistent with this framework, we observed that selenium intake was inversely associated with both RC and TNF-α, whereas RC correlated positively with TNF-α; mediation analysis further showed that TNF-α partially mediated the inverse association between selenium intake and RC in older women. These findings are compatible with an inflammation-related pathway linking selenium status to RC.

Rather than focusing on single nutrients, we derived nutrient patterns using PCA to capture nutrient co-exposure structure relevant to RC. Only the “high selenium-vitamin A” pattern was inversely associated with RC, predominantly in women, suggesting that selenium-centered dietary profiles may represent diet-related correlates of lower RC in older women. By contrast, TNF-α mediation was evident for selenium intake but not for the pattern. A plausible explanation is that PCA-derived scores aggregate multiple nutrients, which may attenuate selenium’s specific inflammatory linkage to RC when other components vary. Moreover, as a data-driven method, PCA is sensitive to variable inclusion and rotation, and these choices can influence associations between nutrient patterns and outcome [35].

Prior evidence indicates sex differences in lipid metabolism: premenopausal women generally have a more favorable lipid profile than men, characterized by lower levels of small, dense LDL particles and higher concentrations of large HDL particles, whereas the abrupt postmenopausal decline in estrogen amplifies systemic inflammation and shifts lipids toward a more atherogenic profile, thereby increasing cardiometabolic risk [36]. Selenium biology also appears sex-sensitive: circulating selenium and selenoprotein P levels often differ by sex (typically higher in men), likely reflecting combined influences of the hormonal milieu, body composition, dietary intake and absorption, and genetic variation, although the precise mechanisms remain to be clarified [37]. Consistent with this context, women in our cohort had lower selenium adequacy and higher RC levels than men, enlarging the detectable exposure gradient and potentially accounting for the more pronounced associations observed in women. Notably, the significant interaction of sex was only confined to the association between selenium and RC; nevertheless, simple-slope analyses indicated a stronger association in women. The lack of significance in other models likely reflects limited power from the modest sample size and measurement error in exposure/biomarker assessment.

To our knowledge, this is the first study to systematically evaluate selenium intake and selenium-centered nutrient patterns in relation to RC in older adults, integrating single-nutrient analyses, PCA-derived patterns, sex-stratified models, and mediation testing. This stepwise approach refines understanding of how selenium-related dietary exposures relate to RC. Our findings highlight that selenium and selenium-centered nutrient patterns are significantly negatively associated with RC, which may inform nutrition-oriented approaches to RC in the older population. Additionally, TNF-α partially mediated the association between selenium intake and RC in women, which may suggest a possible inflammation-related pathway.

However, several limitations should be considered. First, the cross-sectional design allows for association analysis but does not establish causality. Second, the use of a 24-hour dietary recall may not fully capture long-term nutrient intake patterns in the Chinese population; future studies could consider more reliable methods, such as multiple 24-hour recalls or food frequency questionnaires. Third, while the study focused on the older people and included sex-based subgroup analyses, the relatively small sample size and single-region scope may limit the generalizability of the findings. Fourth, the absence of blood nutrient concentration measurements may hinder an accurate assessment of nutritional status. Besides, we lacked data on other key lifestyle factors such as physical activity, sleep, and drinking, which could confound the observed associations. Lastly, we measured only a single inflammatory marker, which limits the interpretation of potential mechanisms. Future research should integrate these factors to provide a more comprehensive assessment of the effects of selenium and other micronutrients on human health.

## Conclusion

In conclusion, micronutrient inadequacies are widespread among older individuals in rural northern China. Selenium, along with selenium-centered dietary pattern, were negatively associated with RC, with selenium potentially exerting its protective effects through inflammation reduction, particularly in older women. These findings suggest that selenium intake adequacy and selenium-centered dietary patterns may inform nutrition-oriented approaches to RC management and CVD prevention in the older people. Large, multicenter prospective studies across diverse regions are needed to confirm these associations.

## Supplementary Information

Supplementary Material 1: Table S1 The micronutrient intakes between different sexes. Table S2 Prevalence of micronutrient inadequacy between women and men. Table S3 Orthogonally rotated factor loadings for the five nutrient patterns^a^. Fig. S1 Four nutrient patterns extracted by principal component analysis. Table S4 The multiple linear regression of selenium intake with RC and TNFα in different groups of sex. Fig. S2. The nonlinear exploration in the associations of selenium intake with RC and TNF-α stratified by sex. Table S5 The multiple linear regression of four nutrient patterns with RC and TNFα in different groups of sex. Fig. S3 Sex interactions in the associations of selenium-related exposures with RC and TNF-α. Table S6 Simple slopes by sex for the associations of selenium intake and the selenium-vitamin A pattern with RC and TNF-α. Fig. S4 Contributions of different foods to selenium intake in our cohort.

## Data Availability

The data that support the findings of this study are available from the corresponding authors upon reasonable request.

## Abbreviations

CVD: Cardiovascular disease
LDL-C: Low-density lipoproteins cholesterol
RC: Remnant cholesterol
TRL: Triglyceride-rich lipoprotein
VLDL: Very-low-density lipoprotein
LDLR: Low-density lipoprotein receptor
PCA: Principal component analysis
NPs: Nutrient patterns
TNF-α: Tumor necrosis factor-α
WC: Waist circumference
SBP: Systolic blood pressure
DBP: Diastolic blood pressure
BMI: Body mass index
HTN: Hypertension
OGTT: Oral glucose tolerance trial
NGT: Normal glucose tolerance
Pre-DM: Pre-diabetes mellitus
DM: Diabetes mellitus
HbA1c: Glycosylated hemoglobin
HPLC: High-performance liquid chromatography
CV: Coefficient of variation
FPG: Fasting plasma glucose
ALT: Alanine aminotransferase
sCr: Serum creatinine
sUA: Serum uric acid
TG: Triglyceride
TC: Total cholesterol
HDL-C: High-density lipoproteins cholesterol
eGFR: Estimated glomerular filtration rate
RNI: Recommended nutrient intake
SD: Standard deviation
CIs: Confidence intervals
IL-1β: Interleukin-1β
IL-6: Interleukin-6
