# Supplementary material for: Selenium intake and a selenium-centered nutrient pattern are inversely associated with remnant cholesterol among older women in rural China: partial mediation by tumor necrosis factor-α
Source: BMC Geriatr. 2025 Dec 23;26:112. doi: 10.1186/s12877-025-06807-7 (PMC12837417; doi:10.1186/s12877-025-06807-7)
Supplement: Supplementary file 1 — Supplementary Material 1: Table S1 The micronutrient intakes between different sexes. Table S2 Prevalence of micronutrient inadequacy between women and men. Table S3 Orthogonally rotated factor loadings for the five nutrient patternsa. Fig. S1 Four nutrient patterns extracted by principal component analysis. Table S4 The multiple linear regression of selenium intake with RC and TNFα in different groups of sex. Fig. S2. The nonlinear exploration in the associations of selenium intake with RC and TNF-α stratified by sex. Table S5 The multiple linear regression of four nutrient patterns with RC and TNFα in different groups of sex. Fig. S3 Sex interactions in the associations of selenium-related exposures with RC and TNF-α. Table S6 Simple slopes by sex for the associations of selenium intake and the selenium-vitamin A pattern with RC and TNF-α. Fig. S4 Contributions of different foods to selenium intake in our cohort. [file 12877_2025_6807_MOESM1_ESM.docx]

**Supplementary Materials**

**Page 1:** Table S1. The micronutrient intakes between different sexes.

**Page 2:** Table S2. Prevalence of micronutrient inadequacy between women and men.

**Page 3:** Table S3. Orthogonally rotated factor loadings for the five nutrient patterns^a^.

**Page 4:** Fig. S1. Four nutrient patterns extracted by principal component nutrient patterns^a^.

**Page 5:** Table S4. The multiple linear regression of selenium intake with RC and TNFα in different groups of sex.

**Page 6：**Fig. S2. The nonlinear exploration in the associations of selenium intake with RC and TNF-α stratified by sex.

**Page 7:** Table S5. The multiple linear regression of four nutrient patterns with RC and TNFα in different groups of sex.

**Page 8:** Fig. S3. Sex interactions in the associations of selenium-related exposures with RC and TNF-α.

**Page 9：**Table S6. Simple slopes by sex for the associations of selenium intake and the selenium-vitamin A pattern with RC and TNF-α.

**Page 10**: Fig. S4 Contributions of different foods to selenium intake in our cohort

**Table S1** The micronutrient intakes between different sexes.

|  | Overall (n=378) | Women (n=210) | Men (n=168) | *P* |
| --- | --- | --- | --- | --- |
| Energy (kcal) | 1305.39 (964.23, 1804.10) | 1131.33 (880.04, 1523.41) | 1594.66 (1163.17, 2174.19) | <0.001* |
| Vitamin A (ug) | 189.77 (77.84, 363.75) | 196.55 (80.68, 368.07) | 184.45 (73.13, 360.67) | 0.609 |
| Vitamin B1 (mg) | 0.67 (0.45, 0.96) | 0.60 (0.42, 0.87) | 0.80 (0.50, 1.07) | <0.001* |
| Vitamin B2 (mg) | 0.50 (0.33, 0.76) | 0.49 (0.29, 0.72) | 0.53 (0.38, 0.83) | 0.014* |
| Vitamin B3(mg) | 8.33 (5.95, 11.84) | 7.41 (4.73, 9.95) | 10.01 (6.84, 14.42) | <0.001* |
| Vitamin C (mg) | 44.17 (22.89, 82.45) | 42.75 (20.51, 78.17) | 45.02 (25.95, 83.82) | 0.363 |
| Vitamin E (mg) | 11.91 (6.85, 20.51) | 10.75 (6.46, 17.81) | 13.04 (8.01, 23.50) | 0.034* |
| Phosphorus (mg) | 662.41 (487.79, 899.18) | 583.07 (430.61, 799.56) | 762.62 (566.30, 1038.39) | <0.001* |
| Potassium (mg) | 1047.65 (713.16, 1537.33) | 945.47 (689.64, 1376.84) | 1236.74 (777.13, 1717.24) | 0.001* |
| Calcium (mg) | 237.07 (141.40, 439.05) | 228.89 (128.49, 420.68) | 264.02 (157.02, 459.44) | 0.093 |
| Magnesium (mg) | 193.62 (144.45, 278.99) | 181.87 (136.41, 248.51) | 228.59 (165.48, 318.31) | <0.001* |
| Iron (mg) | 13.28 (9.60, 18.76) | 11.87 (8.62, 16.47) | 14.96 (11.18, 21.79) | <0.001* |
| Selenium (ug) | 26.32 (18.04, 38.81) | 23.86 (15.73, 31.98) | 32.31 (21.07, 46.92) | <0.001* |
| Zinc (mg) | 6.71 (4.73, 9.30) | 6.02 (4.25, 7.97) | 7.86 (5.91, 10.36) | <0.001* |
| Copper (mg) | 1.26 (0.90, 1.82) | 1.15 (0.80, 1.64) | 1.43 (1.00, 2.03) | <0.001* |
| Manganese (mg) | 3.83 (2.66, 5.39) | 3.44 (2.46, 4.53) | 4.64 (3.15, 6.03) | <0.001* |

**P*<0.05 means statistical difference

**Table S2** Prevalence of micronutrient inadequacy between women and men.

|  | Overall (n=378) | Women (n=210) | Men (n=168) | *P* |
| --- | --- | --- | --- | --- |
| Vitamin A | 349 (92.3%) | 188 (89.5%) | 161 (95.8%) | 0.022* |
| Vitamin B1 | 342 (90.5%) | 193 (91.9%) | 149 (88.7%) | 0.290 |
| Vitamin B2 | 360 (95.2%) | 201 (95.7%) | 159 (94.6%) | 0.627 |
| Vitamin B3 | 307 (81.2%) | 178 (84.8%) | 129 (76.8%) | 0.049* |
| Vitamin C | 310 (82%) | 174 (82.9%) | 136 (81%) | 0.632 |
| Vitamin E | 218 (57.7%) | 130 (61.9%) | 88 (52.4%) | 0.063 |
| Phosphorus | 205 (54.2%) | 137 (65.2%) | 68 (40.5%) | <0.001* |
| Potassium | 324 (85.7%) | 185 (88.1%) | 139 (82.7%) | 0.139 |
| Calcium | 348 (92.1%) | 197 (93.8%) | 151 (89.9%) | 0.160 |
| Magnesium | 307 (81.2%) | 184 (87.6%) | 123 (73.2%) | <0.001* |
| Iron | 129 (34.1%) | 76 (36.2%) | 53 (31.5%) | 0.344 |
| Selenium | 353 (93.4%) | 203 (96.7%) | 150 (89.3%) | 0.004* |
| Zinc | 304 (80.4%) | 166 (79%) | 138 (82.1%) | 0.451 |
| Copper | 80 (21.2%) | 29 (17.3%) | 51 (24.3%) | 0.097 |
| Manganese | 213 (56.3%) | 133 (63.3%) | 80 (47.6%) | 0.002* |

**P*<0.05 means statistical difference

**Table S3** Orthogonally rotated factor loadings for the five nutrient patterns^a^

|  | High Vitamin C/B3-multiple minerals | High Vitamin B1/B2 | Low Vitamin E | High Selenium-vitaminA |
| --- | --- | --- | --- | --- |
| Vitamin A | 0.154 | 0.309 | -0.084 | **0.400** |
| Vitamin B1 | 0.145 | **0.822** | 0.323 | -0.157 |
| Vitamin B2 | 0.004 | **0.798** | -0.24 | 0.279 |
| Vitamin B3 | **0.520** | 0.301 | 0.154 | 0.261 |
| Vitamin C | **0.835** | 0.006 | -0.251 | -0.055 |
| Vitamin E | 0.025 | -0.015 | **-0.701** | -0.174 |
| Phosphorus | **0.807** | 0.141 | 0.243 | 0.295 |
| Potassium | **0.918** | 0.034 | -0.065 | 0.065 |
| Calcium | **0.889** | -0.003 | -0.200 | 0.113 |
| Magnesium | **0.897** | 0.06 | 0.176 | 0.036 |
| Iron | **0.713** | 0.097 | 0.159 | 0.256 |
| Selenium | 0.127 | -0.018 | 0.179 | **0.851** |
| Zinc | **0.798** | 0.194 | 0.23 | 0.265 |
| Copper | **0.605** | 0.107 | 0.278 | -0.023 |
| Manganese | **0.584** | -0.025 | 0.597 | -0.194 |
| Eigenvalue | 6.464 | 1.591 | 1.254 | 1.036 |
| % of Variance | 43.095% | 10.61% | 8.36% | 6.90% |
| Cumulative% | 43.095% | 53.70% | 62.07% | 68.97% |

^a^Factor loadings ≥ 0.47 were considered to have a strong relation with the nutrient pattern.


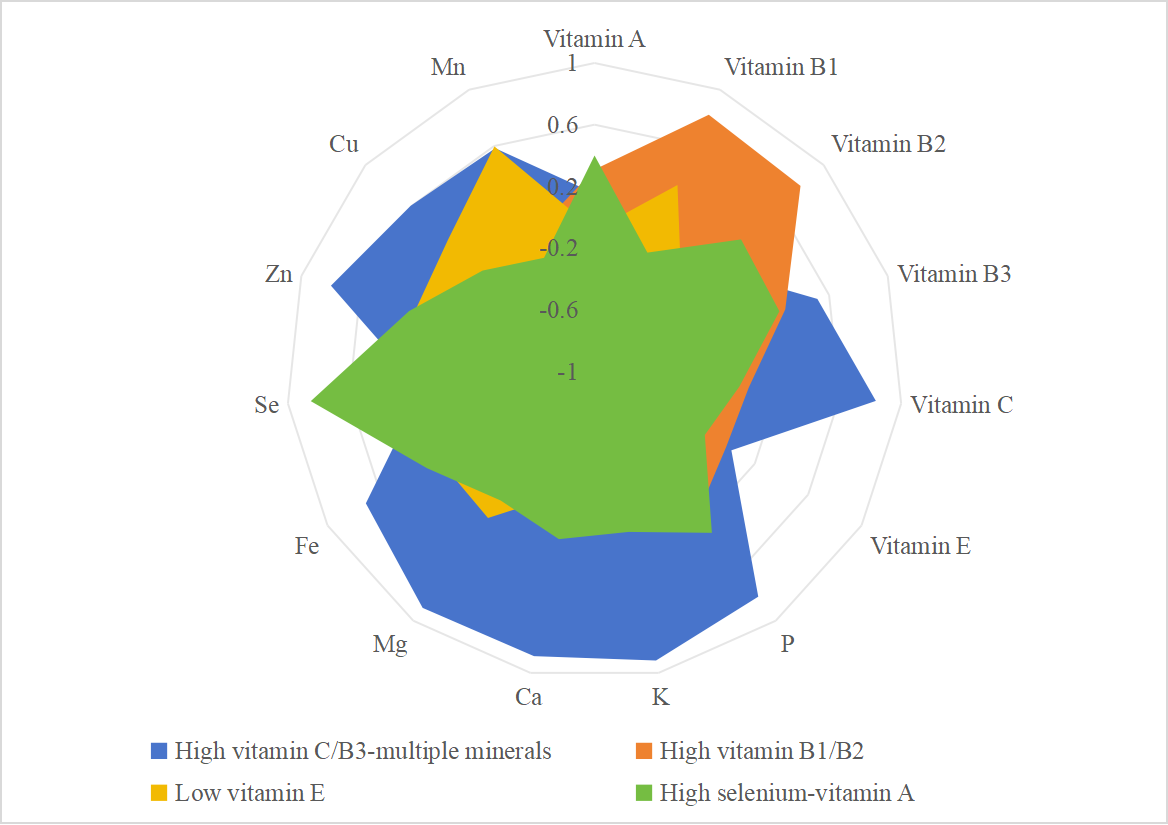


**Fig.S1** Four nutrient patterns extracted by principal component analysis.

**Table S4** The multiple linear regression of selenium intake with RC and TNFα in different groups of sex.

|  | 1 unit increment | | Q1 | Q2 | Q3 | Q4 |  |
| --- | --- | --- | --- | --- | --- | --- | --- |
| RC | β(95%CI) | *P* |  | β(95%CI) | β(95%CI) | β(95%CI) | *P* for trend |
| Women | -0.010  (-0.015,-0.004) | 0.001* | 1(ref) | -0.042(-0.238,0.154) | -0.259(-0.470,-0.049)* | -0.374(-0.587,-0.160)* | <0.001* |
| Men | -0.001  (-0.004,0.001) | 0.356 | 1(ref) | -0.147(-0.353,0.059) | -0.187(-0.368,-0.006)* | -0.266(-0.471,-0.060)* | 0.037* |
| TNFα |  |  |  |  |  |  |  |
| Women | -0.266  (-0.471,-0.060) | 0.012* | 1(ref) | 3.218(-2.470,8.906) | -7.545(-14.571,-0.519)* | -9.898(-16.964,-2.832) | 0.002* |
| Men | 0.059  (-0.191,0.310) | 0.637 | 1(ref) | -2.374(-9.910,5.162) | 2.566(-7.243,12.376) | 0.992(-8.596,10.580) | 0.808 |

The model was adjusted for age, total energy intake, DM, HTN, dyslipidemia, ALT, and eGFR.

**P*<0.05 means statistical difference.

**
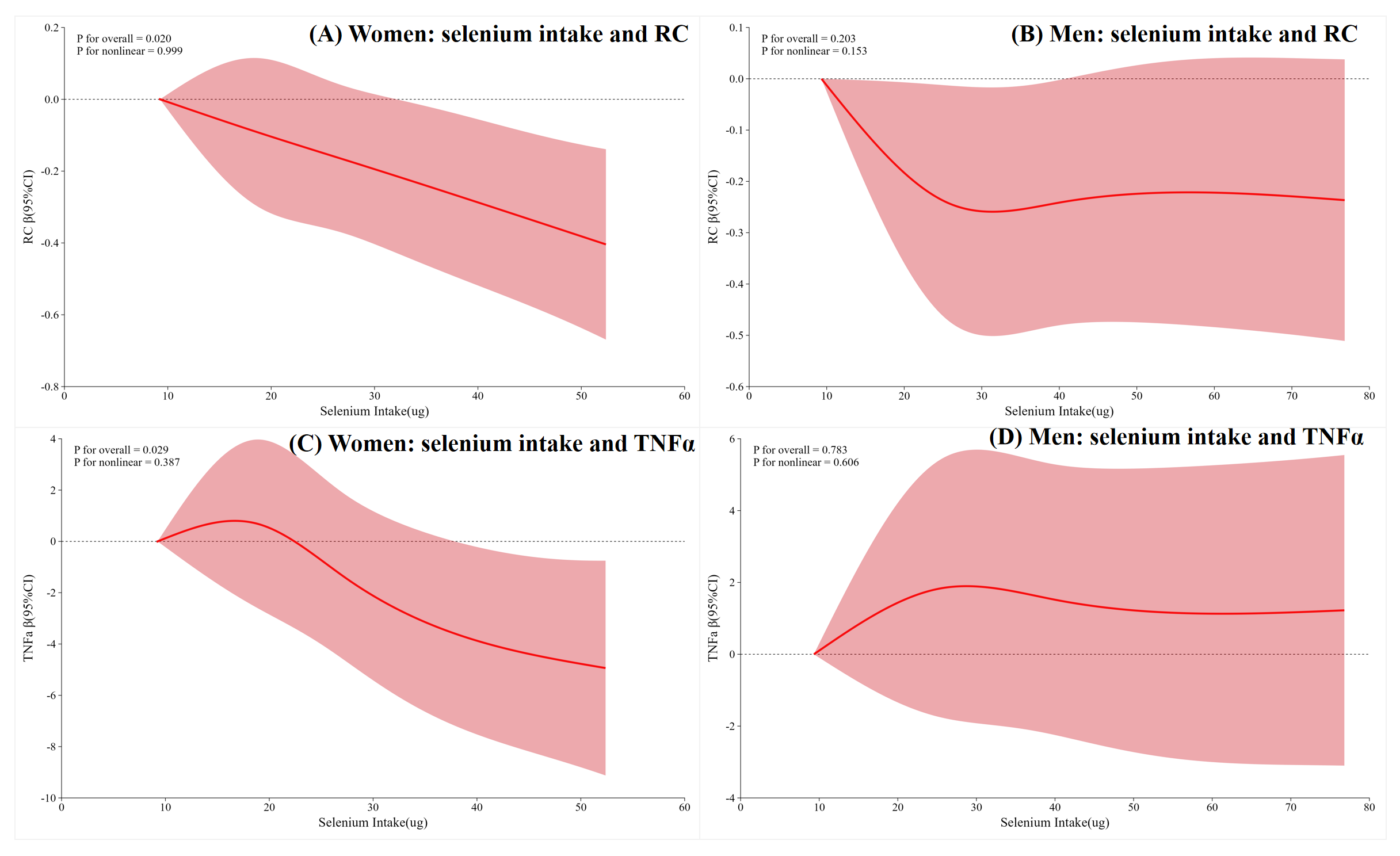
**

**Fig. S2 The nonlinear exploration in the associations of selenium intake with RC and TNF-α stratified by sex**.|(A) Selenium intake with RC in women; (A) Selenium intake with RC in men; (C) Selenium intake with TNF-α in women; (D) Selenium intake with TNF-α in men. The model was adjusted for age, total energy intake, DM, HTN, dyslipidemia, ALT, and eGFR.

**Table S5** The multiple linear regression of four nutrient patterns with RC and TNFα in different groups of sex.

|  | High vitamin C/B3-multiple minerals | | High vitamin B1/B2 | | Low vitamin E | | High selenium-Vitamin A | |
| --- | --- | --- | --- | --- | --- | --- | --- | --- |
| RC | β(95%CI) | *P* | β(95%CI) | *P* | β(95%CI) |  | β(95%CI) | *P* |
| Women | -0.061  (-0.148,0.026) | 0.134 | -0.041  (-0.173,0.091) | 0.485 | -0.012  (-0.089,0.065) | 0.769 | -0.148  (-0.239,-0.058) | 0.001* |
| Men | 0.035  (-0.022,0.092) | 0.253 | -0.016  (-0.089,0.057) | 0.654 | -0.018  (-0.078,0.042) | 0.559 | -0.034  (-0.091,0.023) | 0.240 |
| TNFα |  |  |  |  |  |  |  |  |
| Women | -0.163  (-2.620,2.295) | 0.953 | 1.369  (-3.175,5.912) | 0.567 | 4.148  (1.585,6.712) | 0.002* | -2.433  (-5.502,0.637) | 0.110 |
| Men | -0.771  (-2.758,1.215) | 0.459 | 4.210  (0.492,7.928) | 0.027* | 3.469  (1.230,5.708) | 0.003* | 1.382  (-2.181,4.945) | 0.416 |

The model was adjusted for age, total energy intake, DM, HTN, dyslipidemia, ALT, and eGFR.

**P*<0.05 means statistical difference.

**
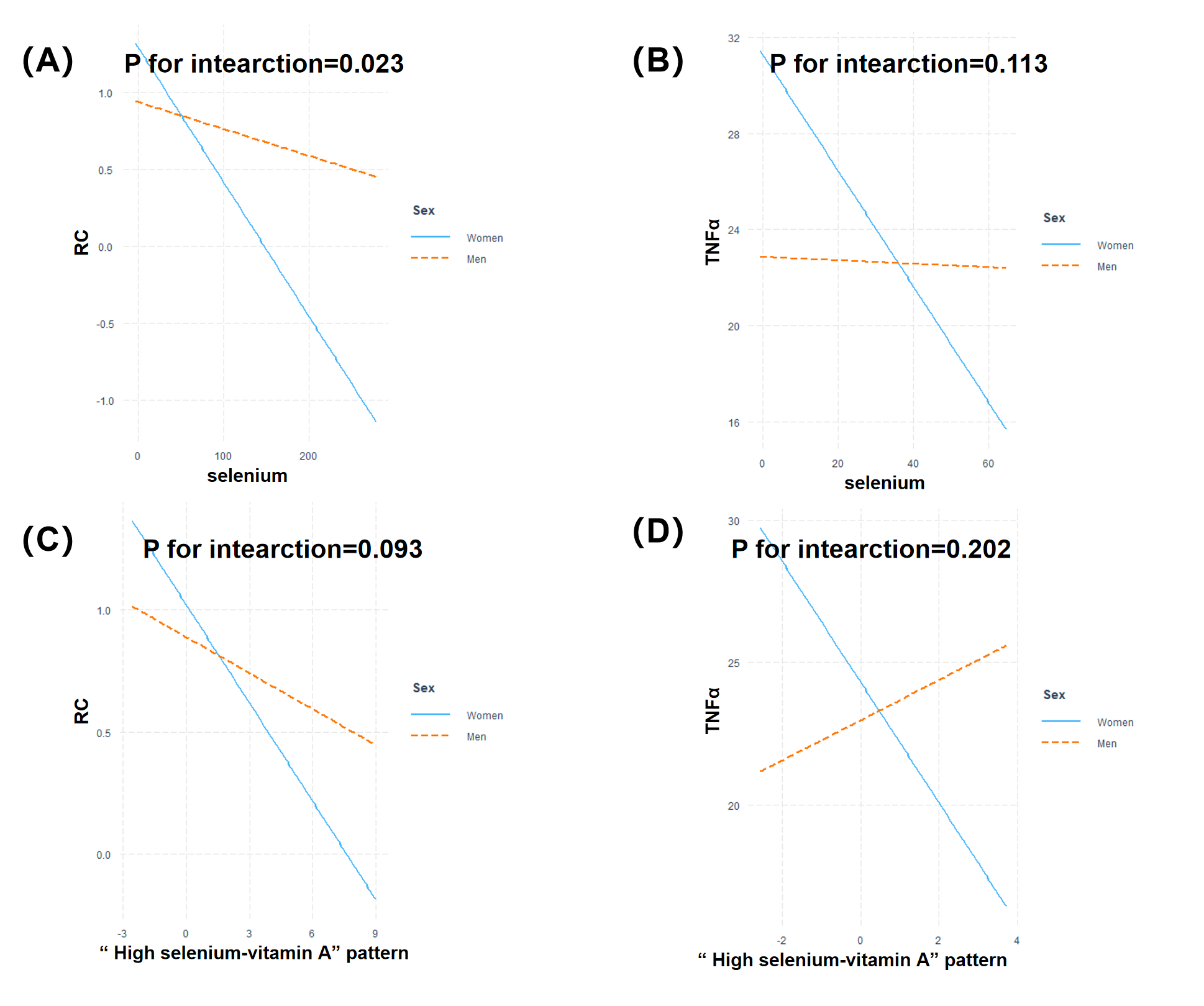
**

**Fig. S3 Sex interactions in the associations of selenium-related exposures with RC and TNF-α**.|(A) Selenium intake with RC; (B) Selenium intake with TNF-α; (C) “high selenium-vitamin A” pattern with RC; (D) “high selenium-vitamin A” pattern with TNF-α.

**Table S6** Simple slopes by sex for the associations of selenium intake and the selenium-vitamin A pattern with RC and TNF-α

| Exposure | Outcome | Sex | β(95%CI) | *P* |
| --- | --- | --- | --- | --- |
| Selenium | RC |  |  |  |
|  |  | Women | -0.009 (-0.014,-0.003) | <0.01* |
|  |  | Men | -0.002 (-0.005,0.001) | 0.24 |
| Selenium | TNF-α |  |  |  |
|  |  | Women | -0.241(-0.437,-0.046) | 0.02* |
|  |  | Men | -0.007 (-0.233,0.218) | 0.95 |
| “high selenium-vitamin A”pattern | RC |  |  |  |
|  |  | Women | -0.134 (-0.216,-0.052) | <0.01* |
|  |  | Men | -0.049 (-0.109,0.011) | 0.11 |
| “high selenium-vitamin A”pattern | TNF-α |  |  |  |
|  |  | Women | -2.115 (-4.990,0.758) | 0.15 |
|  |  | Men | 0.705 (-2.710,4.723) | 0.68 |

The model was adjusted for age, total energy intake, DM, HTN, dyslipidemia, ALT, and eGFR.

**P*<0.05 means statistical difference.


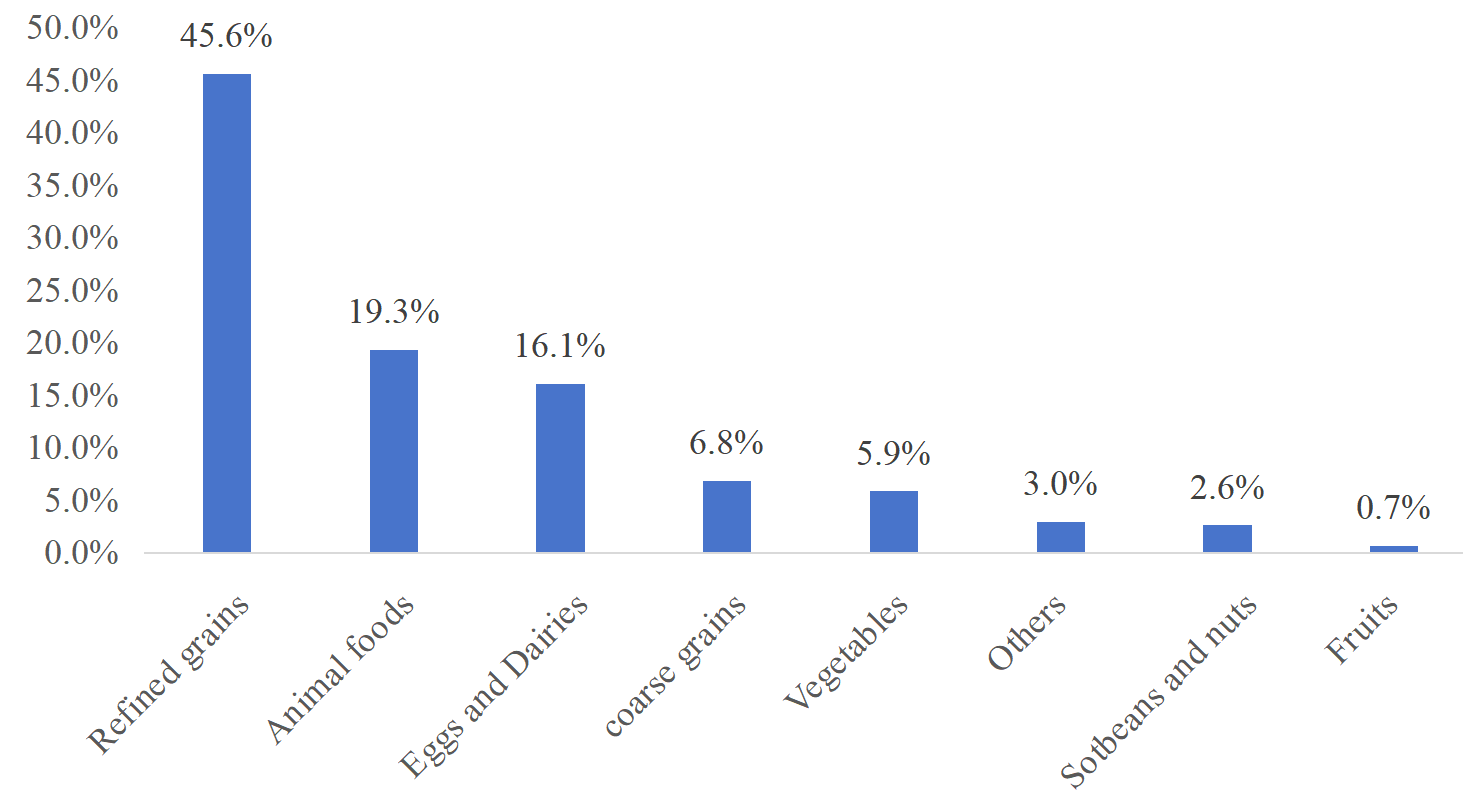


**Fig. S4** Contributions of different foods to selenium intake in our cohort
